# Salinity patterns and local migration determine the isotopic composition of the invasive blue crab, *Callinectes sapidus*, along the Spanish Mediterranean coast

**DOI:** 10.1371/journal.pone.0313429

**Published:** 2025-02-12

**Authors:** Patricia Prado, Iraida Català, Carles Alcaraz, Maria del Carmen Barberà, Elena Guijarro-García, Silvia Falco

**Affiliations:** 1 Instituto de Investigación en Medio Ambiente y Ciencia Marina (IMEDMAR-UCV), Universidad Católica de Valencia SVM, Calpe, Alicante, Spain; 2 Institut d’Estudis Professionals Aqüícoles i Ambientals de Catalunya (IEPAAC), La Ràpita, Tarragona, Spain; 3 IRTA La Ràpita, Tarragona, Spain; 4 Facultad de Ciencias, Universidad de Alicante, Sant Vicent del Raspeig, Alicante, Spain; 5 Instituto Español de Oceanografía, Centro Oceanográfico de Murcia, Lo Pagan, Murcia, Spain; 6 Instituto de Investigación para la Gestión Integrada de Zonas Costeras (IGIC), Universitat Politècnica de València, Gandía, Valencia, Spain; University of Basrah, IRAQ

## Abstract

δ^13^C and δ^15^N patterns of the blue crab, *Callinectes sapidus*, and its potential diets, were investigated in eleven localities within four invaded regions of the Spanish Mediterranean (Catalonia, Valencia, Alicante, and Murcia) subjected to contrasting salinities and degrees of connectivity with the open sea. These regions host blue crab fisheries of variable importance that might be regulated by availability of food resources and local environmental conditions. When present, large adults and immature, subadult sizes of each sex were captured to evaluate possible differences associated to age movement across habitats. SIBER and MixSIAR were used to assess patterns of isotopic niche and dietary contributions. Results showed significant effects for all factors and interactions (except for Sex and Sex x Size in δ^15^N). The effect size in Region (η_p_^2^ = 0.82 and 0.70, respectively for δ^13^C and δ^15^N) and Locality (η_p_^2^ = 0.53 and 0.46), was substantially greater than that of crab Size (η_p_^2^ = 0.37 and 0.21) and Sex (η_p_^2^ = 0.06 and 0.02), concurring with increasing levels of isotopic niche overlap (9% and 11% in Locality and Region, 32% in Size and 44% in Sex). MixSiar results indicated strongly variable contributions from food sources at each locality (TPs from 2.3 to 3.6), but with higher quantity of fish, algae, and crustaceans (27.7%, 18% and 15.1%). Dietary results showed little relation with isotopic patterns, whereas significant associations were found between local salinities and signatures in both crabs (*R*^2^ = 0.518 and 0.757, for δ^13^C and δ^15^N) and diets. Overall, our study suggests that blue crab habitat use in small Mediterranean estuaries might largely differ from native areas, with movements being mostly restricted to young individuals and/ or certain localities with higher connectivity with the open sea (e.g., the Ebro River). Salinity conditions emerge as a major variable shaping isotopic patterns of populations on a large scale.

## Introduction

The blue crab (*Callinectes sapidus* Rathbun) is considered one of the 100 most dangerous invasive species in the Mediterranean Sea, capable of altering the biodiversity and trophic functioning of native communities, and causing economic and social losses to local fisheries, aquaculture, and traditional practices [1–3]. In Spain, the national legislation has not included the species in the Spanish Catalog of Invasive Exotic Species as the only means to market the blue crab and control its spread [4]. Originally native to the western Atlantic Ocean, from Nova Scotia to Argentina [5], the blue crab was introduced to the Eastern Mediterranean Sea in 1948 [6], likely through ballast water discharge from ships or via the Suez Canal. Mancinelli et al. [7] highlighted the ubiquitous spread of the species, with up to 458 records in the Mediterranean Sea, particularly in Spain, Italy, and Greece. Researchers emphasize that an anomalous accumulation of records has occurred in Spain during the last 10 years, given that the first population of the species in the Tancada lagoon (Ebro Delta, Catalonia) was found in 2012 [8], which is comparatively much more recent than those in Greece and Italy dating from the late 40s. In fact, the blue crab has experienced a very quick expansion along the Spanish Mediterranean coast. A single specimen was first detected in 2004 in Mar Menor (Murcia), followed by the Ebro Delta population in 2012, and since then, it has reached the Albufera lagoon (Valencia) in 2013, Gandía also in Valencia in 2014, the Segura River (Alicante) in 2015 [9, 10] and the Balearic Islands in 2017 [11]. In late 2018, the blue crab was found on the Southern Atlantic coast of Portugal [12], despite the absence of estuarine habitats between the Mar Menor and the Gibraltar strait. In fact, recent research by González-Ortegón et al. [13], indicate that only two different haplotypes are present along the Spanish Mediterranean coast, one haplotype being dominant from the Ebro Delta to the Mar Menor, and a second haplotype being more representative of Alboran Sea populations, featuring high connectivity with the Gulf of Cadiz.

The blue crab is regarded as a generalist omnivore, feeding on any available food resource depending on local availability [14]. Hines [15], conducted an extensive review of stomach contents and other feeding observations and indicated that the blue crab diet might include at least 99 species from several phyla, especially mollusks (20–40%), arthropods (10–26%), chordates (fishes; 5–12%) and annelids (polychaetes; 1–7%). Additionally, the diet often includes significant contributions of plant material (1–20%) and algae (3–30%), as well as sediments (up to >50%) when prey items become scarce [16]. Yet, the blue crab might also change during development, with juveniles inhabiting shallower waters feeding on a variety of small epibiota and infauna, and large adults targeting larger and less diverse prey [17, 18]. Miller et al. [19] also indicated that the consumption of bivalves was the highest (39%) for subadult (60–119 mm) and adult sizes (≥120 mm), whereas recruits (≤59 mm) ingested significantly higher proportions of plant matter (10–12%). Sex might be another source of dietary variability, since microhabitat partitioning is often reported in estuarine mating environments, with large males being captured in shallower or lower salinity areas than mature females [20, 21]. Also, the species features sexual differences in claw morphology which further suggest possible variability in dietary habits [22]. After copulation, female blue crabs are renowned for their spawning migration from the upper estuary to higher salinity waters by the mouths of estuaries and coastal areas [23], which involves further movement across habitats with distinctive food resources. Female blue crabs do not often survive after larval release at high salinities [24], whereas males in estuarine waters may continue to molt and grow for 1–3 additional instars (typical large size is 180–200 mm, but some might grow up to >250 mm; [15]). Larval development includes seven zoea instars, followed by a single megalopal stage that return to settle and metamorphose in submerged macrophytes in the lower estuary, and from there juveniles start dispersing upstream, to lower salinity habitats [25].

Stable isotope analyses (SIA) offer a powerful tool to simultaneously assess the dietary behavior of consumers and their migration patterns. The isotopic niche, as analyzed through the Stable Isotope Bayesian Ellipses (SIBER) framework in R, provides a method to compare the isotopic niches of different populations and quantify their dietary overlap based on their stable isotope compositions [26, 27]. Furthermore, δ^15^N and δ^13^C signatures and variability in both isotope data can be used to feed mixing models such as MixSIAR to estimate the proportions of different food sources in an organism’s diet and the associated uncertainty [28, 29]. δ^15^N signatures can also offer an accurate estimate of the trophic position of a species within the hierarchy of local food webs given that a baseline indicator is provided [3, 30]. Additionally, because freshwater signatures of primary producers are typically more depleted in δ^13^C than those in estuarine and marine environments (by 5 to >9‰) [31, 32], isotopic patterns are effectively transmitted through the entire food web across salinity gradients and can provide useful evidence of habitat use [32–36]. In blue crab, SIA has provided valuable information on the origin of δ^13^C from dietary sources across different habitats through ontogenic stages [37]. In invaded areas, it has helped to understand the functional traits and potential impacts of the species on native fauna through estimation of the trophic position and its isotopic niche [3, 38].

In this context, the objective of the present study was twofold. First, we aimed to assess the dietary habits of the blue crab in invaded Spanish Mediterranean regions, including Catalonia, Valencia, Alicante and Murcia. Within each region, we selected different localities featuring contrasting habitats (lagoons, bays, estuaries, open sea, and agricultural drainage channels), with variable salinity and connectivity conditions, in terms of distance from the open sea and/ or presence of regulated gates. Given that most processes affecting the different life-stages of invertebrates are scale-dependent, evaluation at two different spatial scales (ca. 10s vs. 100s of km) might help to understand functional patterns. Second, we aimed to evaluate differences in diet associated to individual age (immature subadults vs. large adult sizes) and sex-related habits. δ^15^N and δ^13^C signatures of blue crabs were used to assess variability associated to the spatial scale and demographic factors, to build the size and magnitude of overlap of the isotopic niches, and to determine the relative influence of salinity in overall patterns. Furthermore, the dietary composition (mixing models) and trophic position (when possible) were assessed in order to validate observed spatial patterns in stable isotopes. Variability in the isotopic signatures between sizes and sexes that could be associated to short-term mobility patterns was also assessed for each locality, taking into account local connectivity features.

## Materials and methods

### Study sites

**Fig 1:**
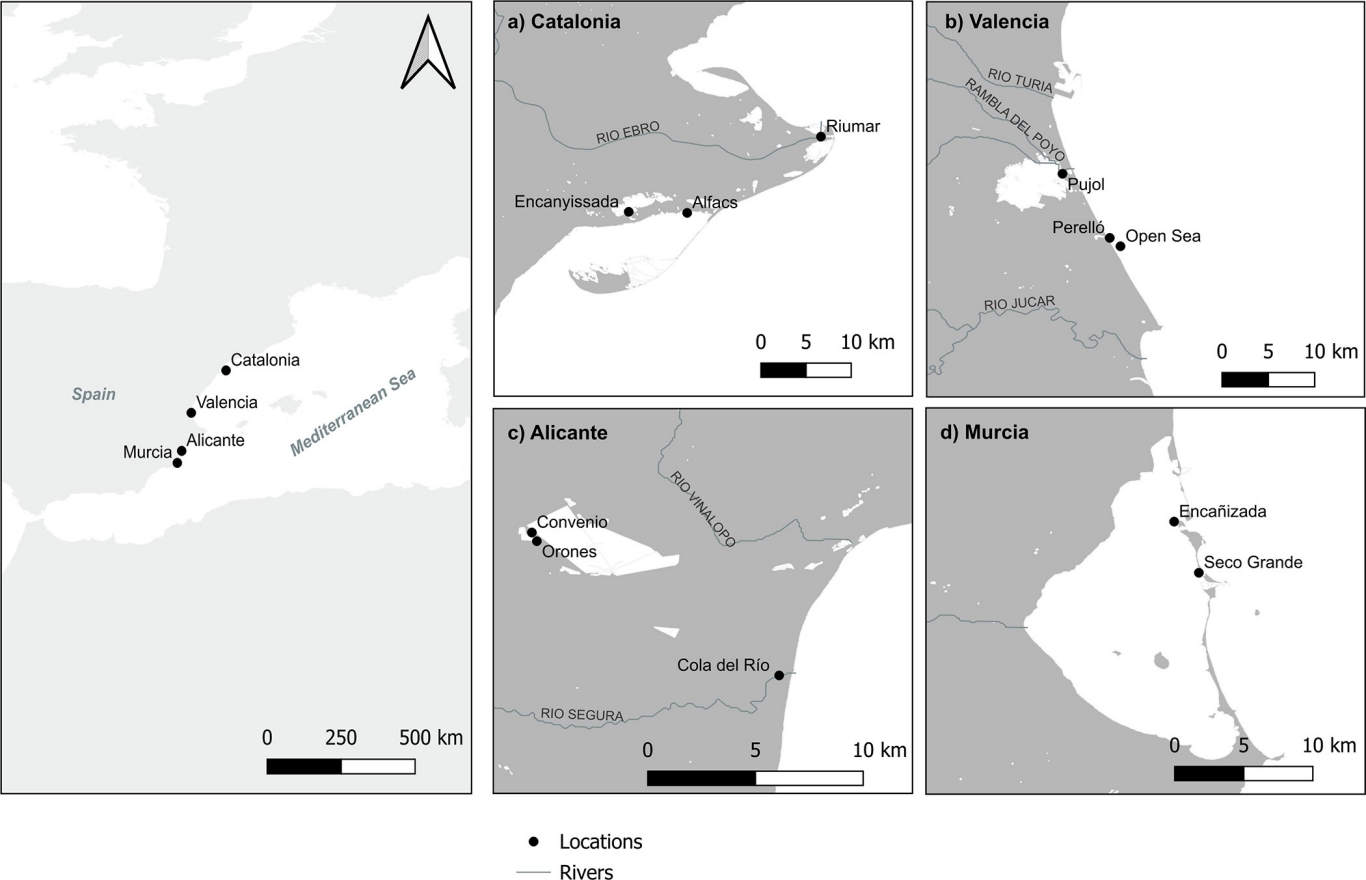
Map of the Spanish Mediterranean coast detailing the position of the four study regions and the localities of blue crab capture. a) Ebro Delta localities (Catalonia); b) Albufera localities (Valencia); c) Segura River and Hondo Lagoon localities (Alicante); and d) Mar Menor lagoon localities (Murcia).

The study was conducted in four regions along the mainland of the Spanish Mediterranean coast: Catalonia, Valencia, Alicante, and Murcia (North to South). Within each region, three localities (only 2 in Murcia) where the blue crab is commonly observed and/or where there are well-established fishery practices targeting the species were selected (i.e., a total of 11 localities). In Catalonia, these localities included (1) an olygohaline to mesohaline stretch of the lower Ebro River between St. Jaume d’Enveja and the Riumar Port; and (2) polyhaline waters of the Encanyissada coastal lagoon, connected to the (3) Alfacs Bay through the Saint Pere channel (Fig 1A) and receiving freshwater discharges from adjacent rice fields that comprise up to 70% of the Ebro Delta surface. In Valencia, localities were selected in the Natural Park of L’Albufera, an area that includes a shallow coastal lagoon and surrounding wetlands connected to the Mediterranean Sea through three one-way gated channels that allow the maintenance of freshwater levels [25]. (4) The Pujol area is within the oligohaline lagoon, whereas (5) the Perelló channel (polyhaline) drains the waters from the Albufera to the open Mediterranean Sea (6) (Fig 1B). In Alicante, localities included (7) Cola del Río at the mouth of the Segura River, and the (8–9) Convenio and Orones channels in the Natural Park of El Hondo located about 10 km inland (Fig 1C), the three of them slightly above the upper limit of the oligohaline stretch. The Natural Park of El Hondo is a wetland system composed of different lagoons and channels, which receives most of its water from the Segura River and aquifer waters, and is surrounded by agricultural lands that have caused severe eutrophication and the disappearance of most of the submerged vegetation following intensive aquifer exploitation and water quality degradation [39, 40]. Finally, in Murcia, two localities were selected in the hypersaline waters of the Mar Menor, the largest coastal lagoon of the Mediterranean basin (Fig 1D). These localities were (10) Seco Grande and (11) Encañizada, both placed in the seaward side of the Mar Menor, further away from areas of agricultural freshwater discharges causing the eutrophication of the lagoon [41]. The Seco Grande is a sand shoal located north of the only navigable channel between the lagoon and the Mediterranean, whereas the Encañizada is a very shallow area with complex water exchange through numerous channels distributed within a lattice of sandbanks and islets.

### Blue crab and food sources sampling

**Ethics statement.** Research conducted in the present study was not experimental. The capture or purchase of commercial fish, blue crabs and other invertebrates in Spain do not require ethics approval and the involvement of an animal research ethics committee. Animals were kept for a couple of hours at -20°C before dissection of muscle tissue.

Blue crabs were captured between mid-June to late August 2022 by deploying either crab traps of different models or gillnets, depending on local fishing gears used across the study regions. At the Riumar locality in the Ebro Delta (Catalonia), where a localized fishery operates in the selected oligohaline to mesohaline stretch of the Ebro River (St. Jaume d’Enveja to Riumar), crabs were obtained directly from the fishermen. The traps were deployed in the morning and collected 24 h later. This operation was repeated as needed to obtain different sexes and sizes. After collection, crabs were put into nylon nets with plastic labels and transported back to the lab in an ice cooler.

At each location we aimed to capture individuals of both sexes within two different size ranges: large adults (120 to 180 mm carapace width with spines), and immature, subadult individuals (50 to 100 mm carapace width, also with spines). However, this was not always possible due to differences in ontogenic and seasonal distribution of crabs, which resulted in certain absences during the study period (see Table 1 for details on the number of individuals of each sex and size obtained at each location). Samples of muscle tissue were consistently extracted from one of the claws and dried at 60 ⁰C until reaching a constant weight. Then they were reduced to powder with a mortar and pestle. Grinding tools were carefully rinsed with ethanol 99% after each sample to remove any remains before reuse. It should be noted that our results are temporally constricted to an estimate isotopic turn-over in blue crab tissues of ca. 3 months [42] during the summer season.

**Table 1:** Number of individuals of each sex and size captured and analyzed for SIA at each locality.

	Locality	Adult ♂	Immature ♂	Adult ♀	Immature ♀
CATALONIA	Riumar	10	13	10	8
	Encanyissada	10	10	10	12
	Alfacs	17	12	10	19
VALENCIA	Pujol	10	10	10	10
	Perelló	7	-	10	8
	Open Sea	-	-	10	-
ALICANTE	Cola del Río	3	-	17	-
	Convenio	12	-	9	-
	Orones	9	-	-	-
MURCIA	Seco Grande	6	7	-	13
	Encañizada	-	11	-	5

Salinity was measured in all localities at the moment of deployment using a multiparametric probe equipped with a data logger, although brands differed among regions. When available (Riumar and Alfacs in Catalonia, and Pujol and Perelló in Valencia), more integrative temporal data across the entire summer period from other ongoing monitoring was also used. When samples were obtained from commercial catches, salinity was measured across the entire fishing ground to obtain spatial averages.

Benthic species of fish and small crustaceans were captured in June-July by deploying three two-meter long fyke nets with 80 cm hoop diameter and 5 mm mesh size were set in shallow areas (60–100 cm depth) in the different localities. Fyke nets were set in late afternoon and hauled the next morning (12 h soaking time) and captures transported to the lab in an icebox for further identification and sorting. Fish and crustaceans were dissected to obtain the musculature and then samples rinsed with ultrapure water to prevent contamination with other tissues.

Bivalve samples of mussel (*Mytilus galloprovincialis*) and oyster (*Crassostrea gigas*) in the Alfacs Bay (Catalonia) were obtained from the local producers. In all localities, infauna (bivalves and Polychaeta) and benthic gastropods were sampled with a hand rake coupled to a net of 0.5 cm mesh. When available, the anemone *Paranemonia cinerea* growing on leaves of *Cymodocea nodosa* was collected by scraping them off with a razor blade. Once in the laboratory, gastropods and bivalves were removed from their shells and all samples rinsed with ultrapure water to remove sediments.

The most abundant species of plant and algae in each locality were either collected by hand or with a Van Veen grab and identified and sorted to species level under the stereomicroscope. The top 2 cm of the sediment layer including an inorganic fraction and detrital material with bacterial/microalgae biofilms was collected (N = 3 per site) using plastic containers. In the lab, samples were homogenized and split in two for the removal of inorganic carbon following the ISO 10694 normative [16].

All animal and vegetal food sources were classified into different groups depending on local availability: fish, crustaceans (crabs and/or prawns), bivalves, gastropods, anemones, odonata larvae, polychaetes, plants, and algae. For each taxon, a minimum of three individuals or vegetation patches was required for inclusion in a given group and considered as an indicator of medium to high availability at the study locality (see S1 Dataset for details on taxa composition). Diet items were dried at 60⁰C, reduced to powder, and weighed. For each locality, an equal quantity of a given taxon (N = 3 for each one collected) was mixed together with other taxa belonging to the same trophic group, to obtain a total of N = 3 replicates (see [16] for a similar approach). Some trophic groups such as anemones and odonata larvae were composed of just one species.

### Stable isotope analyses

Samples were analyzed by isotopic ratio mass spectrometry (IRMS) at the research support service (SAI) of the University of A Coruña, using an elemental analyzer FlashEA1112 coupled with an Interface Conflo II (ThermoFinnigan) to an isotopic ratio mass spectrometer Deltaplus (ThermoFinnigan). Isotope ratios in samples were calculated from linear calibration curves constructed with standard reference materials of known composition and a blank correction. The difference in isotopic composition between the sample and reference material is determined by the equation:

$$\delta_{sample-standard}=[(R_{sample}-R_{standard})/R_{standard}]\;x\;1000$$

where *R*_*sample*_ is the ^13^C/^12^C or ^15^N/^14^N in the sample; *R*_*standard*_ is the ^13^C/^12^C or ^15^N/^14^N in the calibration material and *δ*_*sample-standard*_ is the difference in isotopic composition of the sample relative to that of the reference (Vienna Pee Dee Belemnite for carbon, and atmospheric nitrogen for nitrogen). Experimental precision based on the standard deviation of replicates of acetanilid standard was considered to be adequately high (±0.15 ‰; n = 10).

δ^13^C signatures were normalized for the effect of lipid storage using the equation with C:N ratios indicated by [43] for aquatic animals (corrected δ^13^C = δ ^13^C - 3.32 + 0.99 x C:N), and for vegetal samples (corrected δ^13^C = δ ^13^C + 1.25–0.00 x C:N). Corrections were applied whenever δ^13^C values increased by at least 0.1‰ [44].

### Isotopic data analyses

Differences in the composition of normalized δ^13^C and δ^15^N signatures of blue crabs across Regions (Fixed factor, 4 levels), Localities (Fixed factor, 11 levels, nested in Region), Sex (Fixed factor, 2 levels, nested in Location), and Size (Fixed factor, 2 levels, nested in Location) were investigated with a 4-way mixed ANOVA and SNK post-hoc analysis. The significance of nested factors was assessed through estimated marginal means results. Further effects of Sex and Size at each locality were also assessed with a two-way factorial ANOVA (4 for δ^13^C and 4 for δ^15^N) or one-way ANOVA (5 for δ^13^C and 5 for δ^15^N), depending on samples’ availability.

For all ANOVAs, normality (Chi-square test) and homogeneity of variances (Cochran’s test) were tested. All ANOVA were performed using the GLM package in R. The possible relationships between normalized δ^13^C and δ^15^N in blue crabs and diet groups (N≥ 4 localities) and local salinities were assessed using Pearson’s correlation analysis.

### Blue crab isotopic niche

The package SIBER (Stable Isotope Bayesian Ellipses in R) [26] was used to assess food web structure. Standard ellipses corrected for small sample size (SEAc) were used to represent blue crab samples in the isotopic space. SEAc in units of area (‰^2^) features the same properties of the Total Area (TA) of the convex polygon but it is unbiased with respect to sample size [26]. Then, the Bayesian estimate of the standard ellipse and its area (SEAb) was calculated for each factor (Region, Locality, Sex, and Size) using Markov-Chain Monte Carlo (MCMC) simulations to provide a measure of the uncertainty associated with the standard ellipse area estimate. This method samples randomly replicated sequences in the 95% confidence interval for the value distribution for both stable isotopes (δ^13^C and δ^15^N) to correct the bivariate ellipses. The magnitude of isotopic overlap (‰^2^) across factors levels was obtained from SEAb estimations and expressed as a % of the total area of two given factor levels overlapping each other.

### Estimates of trophic position

The trophic position (TP hereafter) of blue crab was estimated when possible, according to the methodology and equation proposed by [3, 45]:

$${TP}_{\delta 15N}=({\delta^{15}N}_{Consumer}-{\delta^{15}N}_{Baseline})/\Delta^{15}N+\lambda$$

Where δ^15^N Consumer is the nitrogen isotopic signature of the blue crab, δ^15^N baseline is the mean isotopic signature of locally available bivalves as well-known primary consumers (primary producers = trophic level 1, primary consumers = trophic level 2, and so on). Bivalves, and particularly the Mediterranean mussel, have been commonly used as food web baseline in previous stable isotope studies [3, 16, 46–48] and therefore allow for a comparative approach. Δ^15^N is the δ^15^N isotopic enrichment for the blue crab, and λ the trophic level of the baseline indicator, respectively. TP could only be estimated in 4 of the 11 localities (Encanyissada lagoon, Alfacs, Open sea, and Encañizada), were bivalves could be found.

### Isotope mixing models

The contribution of local resources to blue crab diets was assessed with the *MixSIAR Bayesian Mixing Models* (V.3.1) R package [29], using a generalist prior distribution. Models were conducted separately for each locality to avoid possible confounding effects due to large variability in salinity that could strongly influence δ^13^C signatures. For food resources we used the proxy value obtained for each locally available trophic group. For fractionation we applied the 0.4‰ Δ^13^C and the 3.4‰ Δ^15^N values given for non-herbivorous aquatic consumers [30], as they have been largely used for blue crab in previous work [3, 16, 45, 49]. Runs were also conducted using the 1.3‰ SD of δ^13^C and 1‰ δ^15^N indicated by Post [30].

Prior to the application of mixing models, an isospace plot was made to verify that consumer δ^15^N and δ^13^C values fell within the prey polygon in isospace [28]. The Markov Chain Monte Carlo (MCMM) in MixSiar was set as follows: chain length: 100,000; burn-in: 50,000; thin: 50; and number of chains: 3. With these settings, convergence conditions for the Gelman–Rubin diagnostic was <1.05 in all cases; and in the Geweke diagnostic testing for the equality of the means in the first and last part of the Markov chains, the number of variables falling outside the ±1.96 range was < 5% for the three chains. Results corresponding to the 50% quartile were considered as the median source contribution for each diet source along with SD for comparative purposes.

In order to visually assess the similarity between isotopic and dietary patterns at each locality, MixSiar results for median source contributions were squared root transformed and plotted in a nMDS ordination based on Euclidian distances using the PRIMER-6 software package (Primer-E Ltd, Plymouth, UK).

## Results

### Isotopic signatures of blue crab across spatial scales, sex and sizes

**Fig 2:**
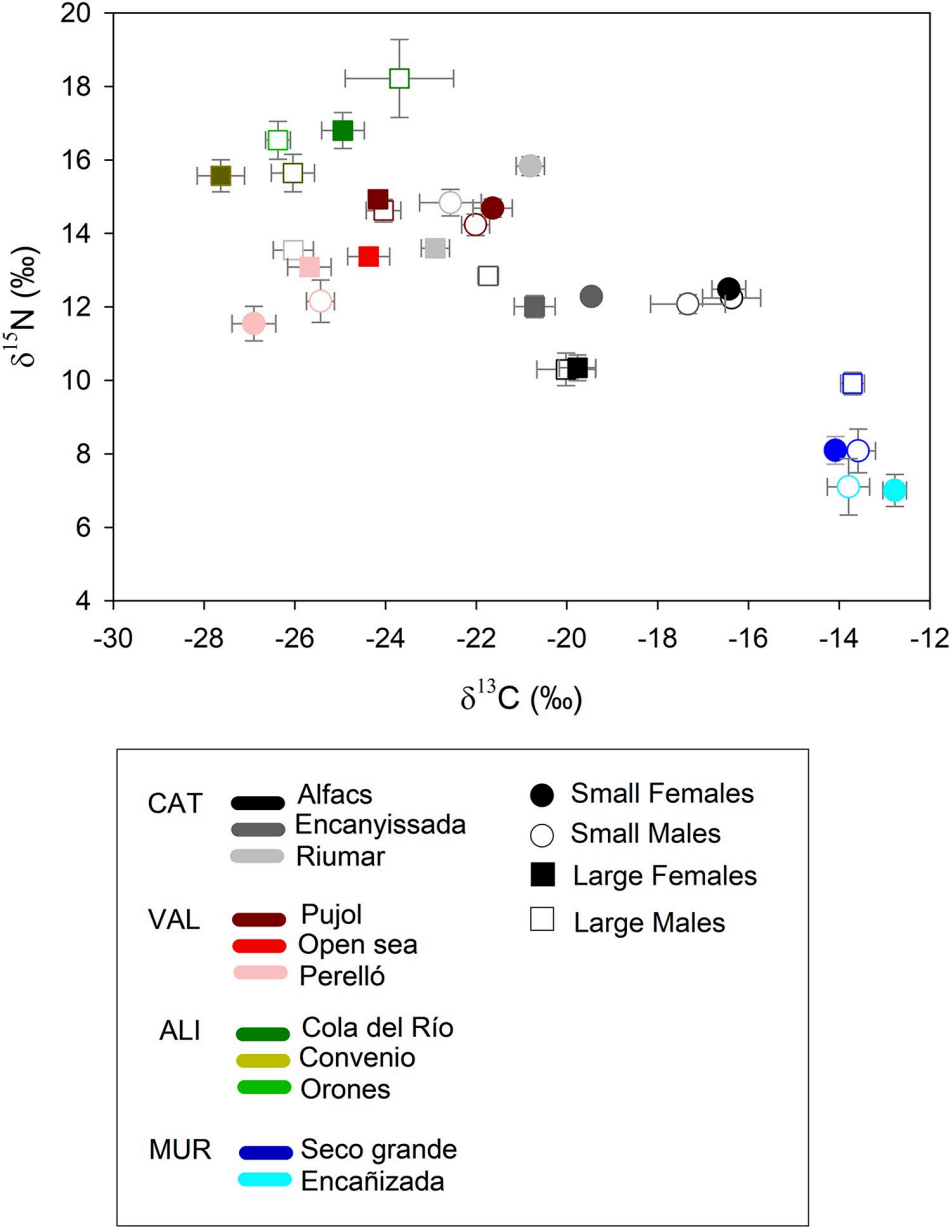
Biplot of δ^13^C and δ^15^N signatures across regions (CAT: Catalonia, VAL: Valencia, ALI: Alicante, MUR: Murcia), localities, sex (male and female), and size (large and small individuals). Error bars are SE.

Patterns of δ^13^C showed significant variability across Regions, Localities, Sex, Size and the Sex- Size interaction, although spatial effects were the most important (Fig 2; Table 2). The highest values were recorded in Murcia (Mur: -13.58 ± 0.25‰), followed by Catalonia (Cat: -20.34 ± 0.13‰), Valencia (Val: -24.28 ± 0.18 ‰) and Alicante (Ali: -25.49 ± 0.37‰). Across Localities, results of estimated marginal means indicated equally high values in Encañizada and Seco Grande (Mur); followed by Alfacs (Cat); the Encanyissada lagoon (Cat); Pujol (Val), Riumar (Cat), and Cola del Río (Ali); the Open Sea (Val); Perelló (Val); Convenio (Ali) and lowest in Orones (Ali). For Sex, males displayed higher values than females (-20.71 ± 0.16‰ vs. -22.28 ± 0.17‰, respectively), and for Size, small immature sizes higher than large adults (-19.56 ± 0.20‰ vs. -23.36 ± 0.12‰, respectively). In particular, the δ^13^C signature of small males was slightly higher than that of small immature females (-17.76 ± 0.27‰ vs. -20.97 ± 0.28‰), and a similar pattern was observed between large adult males and females (-23.01 ± 0.18‰ vs. -23.77 ± 0.17‰).

**Table 2:** 4-way ANOVA of δ^13^C and δ^15^N in blue crab study regions (A = Alicante; V = Valencia; C = Catalonia; and M = Murcia).

δ^13^C						δ^15^N					
Blue crab	df	MS	*F*		*p*	P. eta squared	MS		*F*	*p*	P. eta squared
Region = R	3	1063.46	446.86		**<0.001**	0.828	335.58		222.73	**<0.001**	0.706
Location = L (R)	7	108.70	45.67		**<0.001**	0.535	50.667		33.629	**<0.001**	0.459
Sex = S (L(R))	9	4.85	2.04		**0.035**	0.062	0.957		0.635	0.766	0.020
Size = Si (L(R))	7	55.32	23.24		**<0.001**	0.369	15.916		10.564	**<0.001**	0.210
S x Si (L(R))	5	18.64	7.834		**<0.001**	0.124	1.694		1.125	0.347	0.020
Error	278	2.380					1.507				
SNK(R)				A < V < C < M				M < C < V< A			

Significant values are indicated in bold.

For δ^15^N, results of the 4-way ANOVA were significant for Region, Locality and Size (Fig 2; Table 2). At the regional level, the highest values were observed in Alicante (Ali: 16.40 ± 0.29‰), followed by Valencia (Val: 13.58 ± 0.14‰), Catalonia (Cat: 12.69 ± 0.10‰), and the lowest in Murcia (Mur: 8.03 ± 0.20‰). At local levels, the highest values were detected in Cola del Río (Ali); Orones and Convenio (Ali); Pujol (Val) and Riumar (Cat); Open Sea (Val); Perelló (Val) and Encanyissada lagoon (Cat); Alfacs (Cat); Seco Grande (Mur), and the lowest in the Encañizada (Mur). For Size, a significant enrichment in δ^15^N was observed from small immature individuals (12.43 ± 0.16‰) to large adults (13.73 ± 0.10‰).

2-way ANOVA results of δ^13^C signatures at each locality (Table 3) helped to identify where Size and Sex effects patterns were stronger. In four localities (Alfacs, Encanyissada, Riumar (all three Cat) and Pujol (Val)), small individuals featured higher δ^13^C signatures than large ones, whereas no effects were observed in Perelló (Val), Cola del Río (Ali), Encañizada and Seco Grande (both Mur) and two localities only featured large females (Open sea, Val) or large males: Orones (Ali). Only one locality (Convenio, Ali) featured Sex effects, and a Size x Sex interaction was also observed in the Encanyissada lagoon and in Riumar featuring higher values in small males, and lowest in large males (Table 3).

**Table 3:** ANOVA (1 or 2 way) tests for δ^13^C and δ^15^N in each locality.

Region	Locality		δ^13^C					δ^15^N				
			*df*	MS	*F*	*p-value*	Tukey	*df*	MS	*F*	*p-value*	Tukey
**CATALONIA**	**Alfacs**	Sex (S)	1	0.12	0.03	0.867		1	0.27	0.20	0.654	
		Size (Si)	1	165.59	38.82	**<0.001**	L<S	1	56.55	42.54	**<0.001**	L<S
		S x Si	1	0.33	0.08	0.781		1	0.13	0.1	0.758	
		Residual	54	4.27				54	1.32			
		Total	57					57				
	**Encanyissada**	Sex (S)	1	3.15	1.39	0.246		1	1.05	1.82	0.186	
		Size (Si)	1	83.52	36.74	**<0.001**	L<S	1	0.64	1.11	0.298	
		S x Si	1	25.98	11.43	**0.002**	LM = LF≤SF<SM	1	2.84	4.89	0.053	
		Residual	38	2.27				38	0.58			
		Total	41					41				
	**Riumar**	Sex (S)	1	4.64	2.54	0.120		1	2.19	3.24	0.080	
		Size (Si)	1	76.91	42.05	**<0.001**	L<S	1	30.96	45.72	**<0.001**	L<S
		S x Si	1	59.56	32.56	**<0.001**	LM<LF = SF<SM	1	2.75	4.06	0.051	
		Residual	37	1.83				37	0.68			
		Total	40					40				
**VALENCIA**	**Pujol**	Sex (S)	1	0.16	0.14	0.711		1	1.44	2.06	0.159	
		Size (Si)	1	51.78	44.46	**0.000**	L<S	1	0.99	1.43	0.240	
		S x Si	1	0.62	0.53	0.470		1	0.05	0.07	0.789	
		Residual	36	1.16				36	0.70			
		Total	39					39				
	**Perelló** ^**1**^	Group (G)	2	4.90	2.87	0.078		2	5.42	4.09	**0.031**	SF = LM≤LF
		Residual	22	1.71				22	1.33			
		Total	24					24				
**ALICANTE**	**Cola del Río** ^**2**^	Group (G)	1	14.50	3.77	0.066		1	2.58	0.68	0.418	
		Residual	20	3.85				20	3.78			
		Total	21					21				
	**Convenio** ^**2**^	Group (G)	1	12.94	4.97	**0.038**	LF<LM	1	0.03	0.01	0.915	
		Residual	19	2.60		** **		19	2.53			
		Total	20			** **		20				
**MURCIA**	**Seco Grande** ^**3**^	Group (G)	2	0.67	0.51	0.608		2	7.68	5.57	**0.011**	SM = SF<LM
		Residual	23	1.33				23	1.38			
		Total	25					25				
	**Encañizada** ^**4**^	Group (G)	1	3.58	2.00	0.179		1	0.03	0.01	0.935	
		Residual	14	1.79				14	4.93			
		Total	15					15				

Sex (F = female; M = male), Size (S = small; L = large). One-way ANOVAs with locally available sizes and sexes are indicated with superscripts 1–4. The two localities where only large females (Valencia, open sea) or large males (Alicante, Orones) were available are not indicated. Significant p-values are indicated in **bold**.

^1^ Small males absent, ^2^ Small individuals absent, ^3^ Large females absent, ^4^ Large individuals absent.

For δ^15^N, significant Size or Sex effects were only observed in four localities: Alfacs and Riumar (both Cat), Perelló (Ali), and Seco Grande (Mur) (Table 3). Despite an overall effect of Size (Table 2), with significantly higher values in large individuals, this trend was only observed in Perelló (Val) and Seco Grande (Mur), whereas Alfacs and Riumar (Cat) featured opposite effects, with significantly higher values in immature sizes.

**Fig 3:**
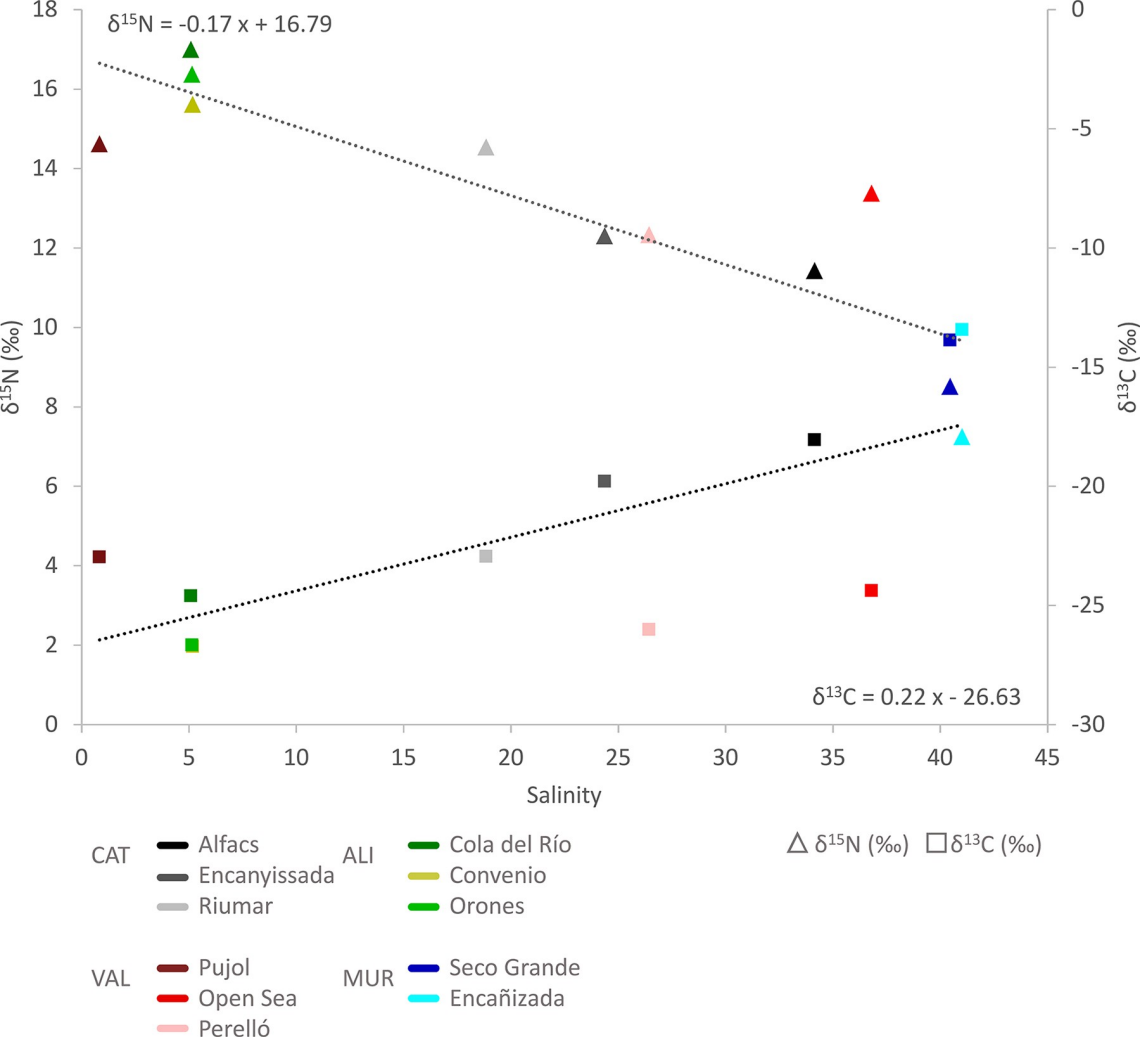
Relationship between δ^13^C and δ^15^N and salinity at each study region. See text for details on *R* and *p* values.

A significant association between isotopic signatures of blue crab and salinity was found for both δ^13^C (df = 10, *F* = 9.69, *R*^2^ = 0.518; *p* = 0.012) and δ^15^N (df = 10, *F* = 28.03, *R*^2^ = 0.756; *p* = 0.0005) (Fig 3), consistent with the spatial aggregation of samples observed in Fig 2. Similar but more inconsistent patterns across isotopic signatures were found for some diet groups (pooled taxa), including sediments (δ^15^N: df = 10, *F* = 50.61, *R*^2^ = 0.849; *p* = 0.00005), plants (δ^13^C: df = 7, *F* = 10.64, *R*^2^ = 0.63; *p* = 0.0173; δ^15^N: *F* = 17.65, *R*^2^ = 0.74; *p* = 0.0056), fish (δ^15^N: df = 10, *F* = 9.39, *R*^2^ = 0.510; *p* = 0.0134), and bivalves (δ^15^N: df = 3, *F* = 22.50, *R*^2^ = 0.918; *p* = 0.0416), but not in algae (df = 6), crustaceans (df = 7), and polychaeta (df = 3).

### Isotopic niches of blue crab

**Fig 4:**
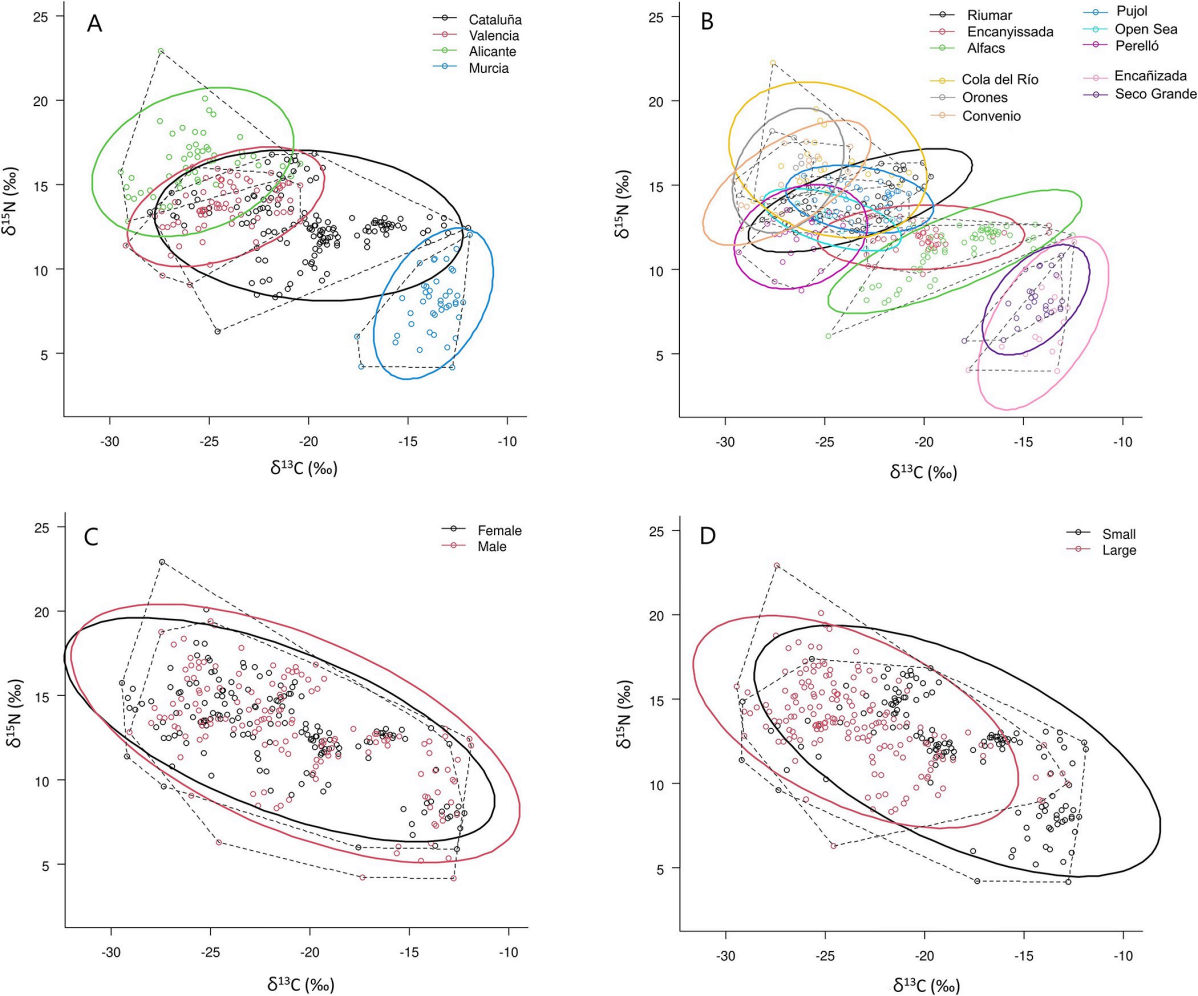
Isotopic niche of *C*. *sapidus* for the different study factors: A) Regions; B) Locality; C) Sex; and D) Size. Points: individual δ^13^C and δ^15^N measurements, dashed lines: total area of the convex hull, colored solid lines: standard ellipse areas corrected for small sample size (SEAc).

**Fig 5:**
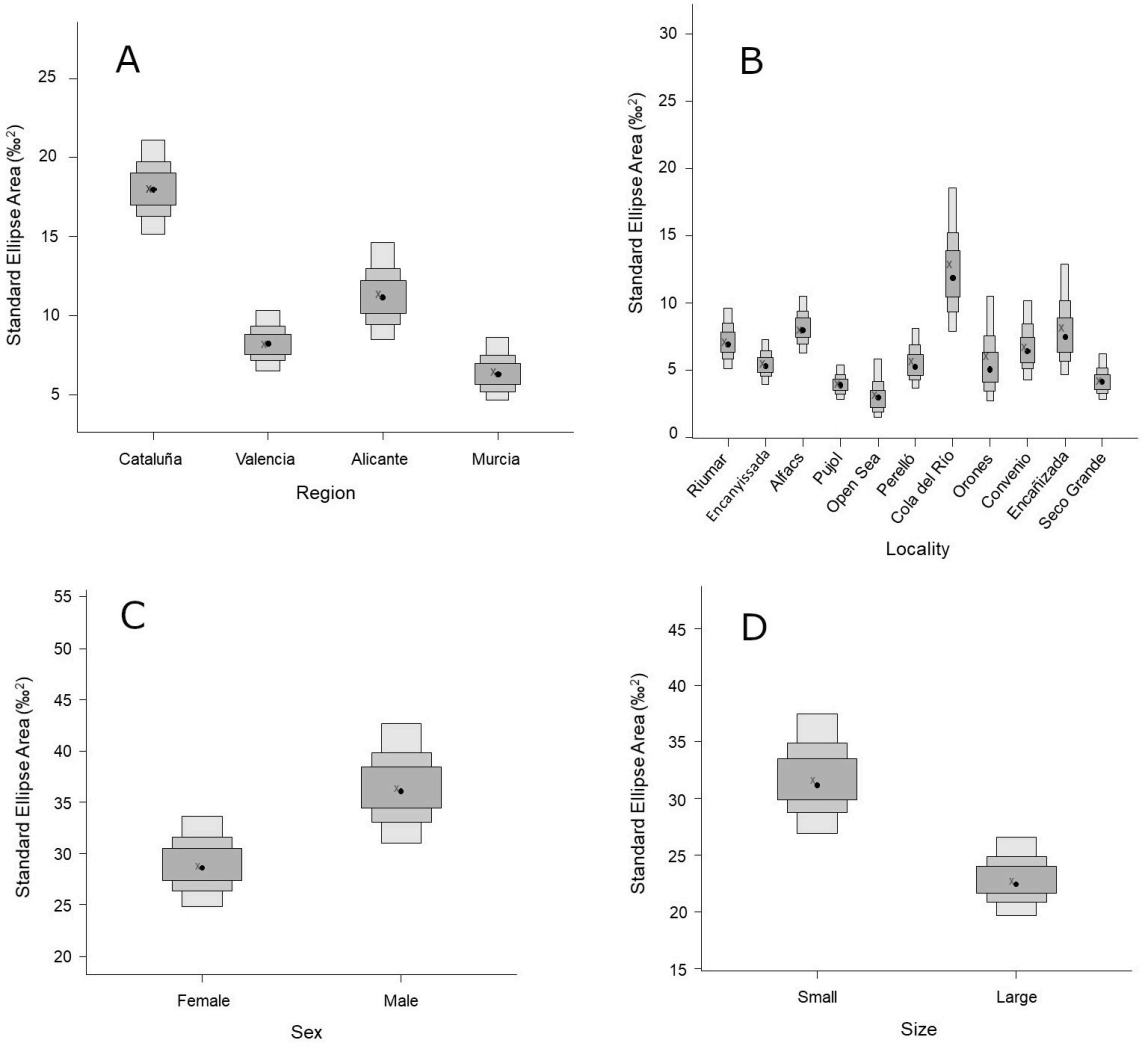
Boxplots of the SIBER Bayesian ellipse areas (SEAb in ‰^2^) of blue crab for the different study factors: A) Region; B) Locality; C) Sex; and D) Size. 50, 75 and 95% credibility intervals are indicated, with dots representing the modes of the Bayesian distributions. The cross indicates the standard ellipse areas calculated using the algorithm for small sample sizes (SEAc).

Bivariate standard ellipses representative of the isotopic niche of blue crab (size-corrected SEAc) also indicated significant variability in distribution and size across Regions, Localities, Sex, and Sizes (Fig 4; Table 4). Among Regions, Catalonia featured the largest size, followed by Alicante and Valencia, and the lowest in Murcia (36.4%, 54.1%, and 63.9% smaller, respectively), although maximum and minimum sizes were attained in Cola del Río (Ali), and the Open Sea (Val)(Fig 5A, 5B). For Sex and Size, males featured larger isotopic niche than females (by 20.7%), and small immature individuals larger than large adults (by 27.8%) (Table 4, Fig 5C, 5D).

**Table 4:** Summary table of isotopic niche metrics for each factor provided by the SIBER package.

		TA	SEA	SEAc
**Region**	Catalonia	91.89	17.99	18.12
	Valencia	38.24	8.20	8.31
	Alicante	47.59	11.25	11.48
	Murcia	26.77	6.38	6.54
**Locality**	Riumar	21.09	7.01	7.19
	Encanyissada	22.12	5.48	5.61
	Alfacs	32.66	7.97	8.11
	Pujol	12.67	3.97	4.07
	Open Sea	5.86	2.91	3.27
	Perelló	18.37	5.55	5.79
	Cola del Río	35.59	12.35	12.97
	Orones	10.79	5.46	6.14
	Convenio	18.71	6.49	6.83
	Encañizada	18.27	7.72	8.27
	Seco Grande	12.71	4.18	4.36
**Sex**	Female	170.12	28.86	29.04
	Male	174.92	36.37	36.62
**Size**	Small	158.15	31.64	31.87
	Large	148.37	22.86	22.99

TA: total area of the convex polygon; SEA: the standard ellipse area; SEAc: standard ellipse area corrected for small sample size.

The significance of the ellipses overlap varied notably among study factors (see S2 Dataset). For Region there was an average overlap of 11.08% considering the total area of any combination of two ellipses, with maximum rates between Cataluña and Valencia (26.47%), and between Valencia and Alicante (22.43%) and no overlap between Murcia and Alicante and Valencia (Fig 4A). For Locality the average overlap was also low (9.35%), but it ranged from 31.47% (between Encañizada and Seco Grande in Murcia) to 31.03% (between Pujol (Val) and Riumar (Cat)), to no overlap between Riumar (Cat), Valencia, and Alicante localities, and the two localities in Mar Menor (Mur) (Fig 4B). The magnitude of overlap was the greatest for Size (32% between subadult and adult sizes), and Sex (44% between males and females) (Fig 4C, 4D), consistent with the size of partial eta squared values in the 4-way ANOVA.

### Trophic position and MixSiar model

The trophic position (TP) of blue crab could only be estimated in 4 of the study localities (Alfacs, and the Encanyissada lagoon in Catalonia, Open Sea in Valencia, and Seco Grande in Murcia) where bivalves could be found and used as a baseline for the calculation. In the Alfacs Bay, the TP of small individuals was 1.26, whereas in Seco Grande the opposite pattern was observed with large individuals featuring 1.21 times higher TP than small individuals, and no clear differences were observed in the Encanyissada lagoon (Table 5). Overall, the highest TP was detected in the Open Sea, whereas in the other localities values varied from 2.4 in the Encanyissada lagoon, 2.58 in the Alfacs Bay (both Cat), and 2.68 in Seco Grande (Mur). These values are slightly lower than those previously recorded in Catalonia using the same methods, particularly for the open sea (Table 5).

**Table 5:** Results of blue crab trophic position (TP) for the different regions, sexes, and sizes investigated in this study.

Region	Site	Sex	Size	Blue crab TP
*Catalonia*	Alfacs Bay^16^	M	L	3.06 ± 0.03
	Tancada lagoon^16^	M	L	2.64 ± 0.21
	Open sea^49^	F	L	4.40 ± 0.06
	Alfacs Bay	M	S	2.84 ± 0.04
		F	S	2.91 ± 0.03
		M	L	2.27 ± 0.13
		F	L	2.28 ± 0.10
	Encanyissada	M	S	2.33 ± 0.07
		F	S	2.39 ± 0.04
		M	L	2.55 ± 0.07
		F	L	2.31 ± 0.09
*Valencia*	Open sea	F	L	3.63 ± 0.07
*Murcia*	Seco grande	M	S	2.50 ± 0.17
		F	S	2.51 ± 0.09
		M	L	3.04 ± 0.09

Data are only shown for those sites in which bivalves were available. For the Catalonian region, TP values previously found in the Alfacs Bay and other habitats of the Ebro Delta are also shown [16, 49] for further comparison.

**Fig 6:**
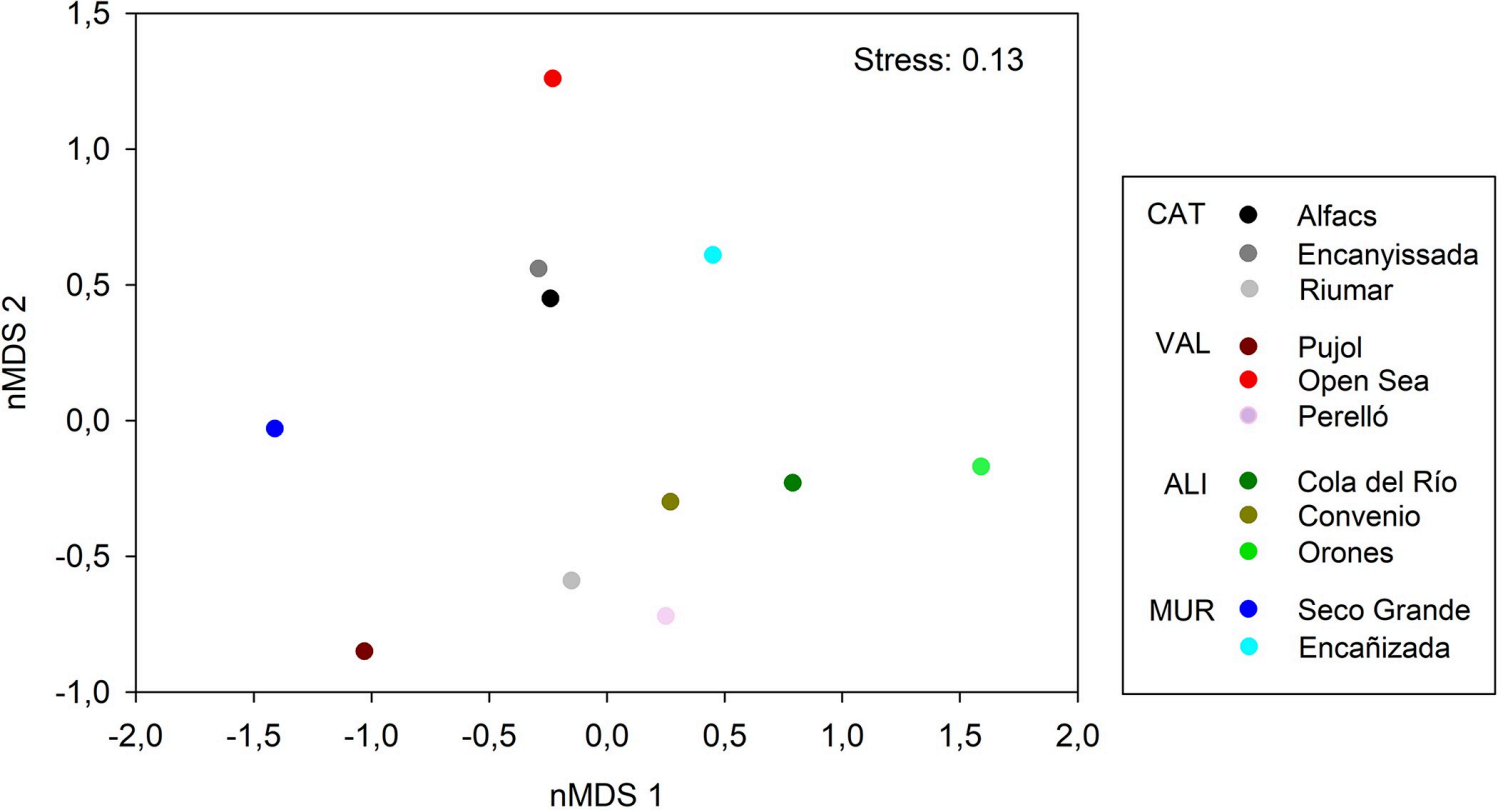
nMDS of median MixSiar results from Table 6 at each locality.

MixSiar model results showed that fish, algae, and crustaceans were the primary food items across locations (Table 6), with average contributions of 25.7%, 18%, and 15.1%, respectively. Other items with contributions between 5 to 7% were plants, sediments, Polychaeta, and bivalves, whereas Actiniaria and Odonate larvae were constricted to certain locations and could be considered occasional items (Table 6). Importantly, however, similarities in dietary composition across localities plotted in nMDS (Fig 6) did not appear to mirror those previously described for isotopic patterns (e.g. the two localities in Murcia are positioned far apart in the nMDS but feature very similar isotopic composition, and a similar incongruence is observed for Open Sea and Pujol (both Val), among others; see Figs 2 and 6).

**Table 6:** MixSiar results (Median value ± SD) showing the % relative contributions of food resources to blue crab diet at each locality.

	Site	Actiniaria	Odonat larvae	Polychaeta	Bivalves	Gastropods	Crustaceans	Fish	Algae	Plants	Sediments
**Catalonia**	Riumar	-	-	-	-	-	34 ± 17	30 ± 18	2 ± 5	10 ± 8	4 ± 3
	Encanyissada	9 ± 10	-	10 ± 10	9 ± 10	6 ± 8	4 ± 6	6 ± 8	16 ± 19	6 ± 10	8 ± 9
	Alfacs	4 ± 8	-	5 ± 8	14 ± 14	3 ± 4	3 ± 6	4 ± 9	31 ± 17	13 ± 8	4 ± 6
**Valencia**	Pujol	-	9 ± 8	-	-	-	56 ± 23	9 ± 12	-	7.3 ± 8	12 ± 17
	Open Sea	-	-	45 ± 19	7 ± 9	-	6 ± 8	22 ± 17	-	5 ± 8	3 ± 6
	Perelló	-	-	-	-	-	20 ± 16	17 ± 12	52 ± 20	-	5 ± 5
**Alicante**	Cola del Río	-	-	-	-	-	-	45 ± 28	26 ± 24	-	16 ± 22
	Orones	-	-	-	-	-	-	92 ± 23	-	-	8 ± 23
	Convenio	-	-	-	-	-	9 ± 10	42 ± 15	16 ± 18	16 ± 13	6 ± 8
**Murcia**	Encañizada	-	-	20 ± 15	-	-	-	11 ± 10	37 ± 23	9 ± 9	7 ± 8
	Seco Grande	-	-	-	28 ± 2	5 ± 8	34 ± 21	5 ± 7	-	12 ± 10	5 ± 7

We used the Δ^15^δN of 3.4 and a Δ^13^δC of 0.4 given for non-herbivorous aquatic consumers [30], and previously used in blue crab [3, 16, 45, 49].

## Discussion

In spite of the large spatial scale evaluated along Spanish Mediterranean coast (ca. 500 km), our results show that stable isotope patterns are strongly driven by freshwater discharges [50]. Blue crab signatures were more depleted in δ^13^C and more enriched in δ^15^N at low salinities, and contrariwise at high salinities, consistent with findings reported in other ecological studies (e.g., [32–36, 51]) and with observations in dietary resources, particularly for δ^15^N. Hence, rather than distance, observed effects of Region and Locality were largely driven by spatial differences in freshwater supply, with Alicante being the region with more oligohaline environments and Murcia the only region with hypersaline sites [40, 52]. For δ^13^C, the effect of salinity is related to a progressive shift in the contributions of organic matter derived from more depleted C3 freshwater macrophytes (e.g., *Potamogeton pectinatus* in Convenio, Pujol, and Riumar) and phytoplankton (<-25 to -19‰), compared to that derived from more enriched C4 salt-marshes, seagrasses (e.g., *C*. *nodosa* in Mar Menor, Alfacs Bay, and the Open Sea), and microalgae in estuarine sites (-18 to -12‰) [31, 32]. In fact, a significant association between δ^13^C and salinity was found for plant resources, whereas for the other dietary groups, the trend was not significant, possibly because they included a pool of different vagile animal species, drifting algae or had low replication (e.g., bivalves). For δ^15^N, high signatures have been commonly associated to intensive human uses and eutrophication of waterways [34, 53]. Several of our study localities have been reported to be impacted by agrochemical and/or urban sewage eutrophication leading to serious degradation in the recent literature: the ecological collapse of the Mar Menor lagoon in 2016 [54], long-term alternations of hydrological and trophic conditions in the Ebro Delta and the Albufera lagoon associated to rice agriculture [55, 56], and severe eutrophication and vegetation loss in river basins of the Alicante region [39]. However, based on the capacity of δ^15^N to act as an indicator of eutrophication in aquatic ecosystems [57], our results suggest that the degree of the environment degradation is the highest in Alicante (16.4‰) followed by Valencia and Catalunya (13.6 and 12.7‰, respectively), and finally by Murcia (8.0‰) localities. In fact, the association of blue crab δ^15^N with salinity levels was even higher than for δ^13^C (by 1.46 times), and significant associations were also found for sediments, plants, bivalves, and even fish, supporting that it is a good approach to the estimation of wastewater inputs. Drifting algae and small crustaceans (amphipods and isopods) also associated with drifting vegetation were the only dietary groups (N≥4) that did not display an association with salinity.

Patterns of isotopic niche were consistent with ANOVA results for both isotopes, and with the effect of the salinity gradient. Spatial overlaps among standard ellipse areas corrected for small sample size (SEAc), indicated Murcia featuring no overlap with other regions except for Catalonia (only 5%), and values from 12% (Catalonia with Alicante) to 26% (Catalonia with Valencia) were observed among the other regions. Yet, in the presence of strong environmental gradients, these differences might be due to feeding at different trophic levels. In contrast, results of relative contributions of food resources to blue crab diet at each Locality from MixSiar did not follow the same patterns of spatial aggregation than those of the δ^13^C and δ^15^N, suggesting some possible caveats in the outputs of model runs associated to various factors. First, dietary assessment was conducted with food resources collected only once, but that might feature significant variation in seasonal isotopic signatures as reported for organic matter sources, macrophytes, invertebrates with a short life cycle, and foraging fish [50, 53]. Additionally, although the model points to an overall larger contribution of fish, algae and crustaceans (25.7, 18 and 15.1%, respectively), particularly at certain regions (e.g., 42 to 92% of fish in Alicante), other resources might have been locally available in the shorter term and unaccounted at the moment of sampling. Second, the enrichment factor of blue crab under natural field conditions may only be approximated, since it can change considerably with the type of diet (e.g., [42, 44, 58], and the species features a remarkable dietary plasticity depending on resource availability [16, 17]. Trophic position, however, can provide a robust diagnostic performance of the trophic ecology of the species, but adequate estimation relies on the availability of primary consumers such as bivalves [3, 16, 45, 46, 49], which in our study were limited to 4 of the 11 localities. According to our results, the Open Sea locality adjacent to the Albufera Lagoon (Valencia) is the site featuring the highest TP (3.63), whereas values ≤3, were observed in the other three localities (Alfacs, Encanyissada, and Seco Grande). Despite a limited number of localities with TP, patterns were neither consistent with differences in isotopic niche (the Open Sea in Valencia was more distinctive than Seco Grande in Murcia) or MixSiar results displaying Polychaeta (deposit-feeding species among others, with a TP of ca. 2; [59, 60]) as the main dietary item in the Open Sea (45%). Nevertheless, enhanced TP in the open sea of Valencia, is consistent with a large TP of 4.40 recently reported for the Open Sea of the Ebro Delta (similar also to other local predatory fish; Prado et al., 2024), suggesting that blue crabs in open waters have access to higher-order consumers than in confined waters, estuarine, or riverine environments. Additionally, current values of TP appear significantly lower (ca. 9 to 16%) than those reported in previous estimates in the Alfacs Bay and adjacent coastal lagoons [16], suggesting a progressive decrease in the availability of higher quality preys. Overall, isotopic niche analysis provided a good indication of aquatic ecosystem type and its inherent variability (i.e., trophic niche width), whereas the TP estimated with a baseline standardization that removes the influence of salinity allowed for certain comparison across ecosystems and attained similarly lower values in confined and semi-confined waters than in the open sea [16, 49].

Subadult, immature blue crab sizes were only captured in six out of the eleven study localities. Four of them (those in Catalonia and Pujol in Valencia, in which all sexes and sizes were present), showed significantly more enriched δ^13^C signatures in subadult sizes compared to large individuals, and similar higher values were also observed for δ^15^N in Alfacs and Riumar, which could be attributed to ontogenic differences in habitat use and access to food resources. In a previous study by Hoeinghaus and Davis [61], authors showed that the δ^13^C signature of blue crabs ranging from 35 to 165 mm carapace width increased significantly with body size due to greater assimilation of carbon ultimately derived from saltmarsh C4 plants. Similar results were also found by [37, 62] who found that juveniles got enriched in δ^13^C as they age and undergo a gradual shift from a planktonic to a benthic-based diet in marsh areas with *Spartina alterniflora*. This meshes well with juvenile blue crabs often being abundant in shallow oligohaline and tidal freshwater marshes, which also appear to offer certain osmotic advantage for enhanced molt increments [63] and refuge from predators [64]). Yet, since individuals >70 mm are indicated to have diets similar to adult sizes [15], the absence of significant differences in δ^13^C signatures is also plausible, although this was observed in the two localities with an incomplete replication (Perelló in Valencia and Seco Grande in Murcia). In contrast, higher δ^13^C values (2.6 to 3.5‰ in Catalonia, and 2.4‰ in Pujol, Valencia) in smaller sizes differ from previous literature trends, and suggest that subadults captured in these localities have spent longer time in higher salinity habitats feeding on more enriched food sources. Although early blue crab instars of 2–3 mm have been detected in the Alfacs Bay (unpublished data) and in Valencian sites using larval collectors [25], the proportion of those arriving at larger stages is unknown and could contribute to significantly more enriched signatures in subadults (although more consistently for δ ^13^C than for δ^15^N). In the Ebro River, recurrent recent droughts [65] may have increased the extent of the salt wedge thus facilitating the arrival of more enriched juveniles from the open sea. In the Alfacs Bay, summer salinity patterns become more diverse [66] due to the effect of rice cultivation discharges, so juveniles from other areas of the bay could have been attracted to our sampling site, and a similar pattern might have been at play in the Encanyissada lagoon. In contrast, in Pujol, an area of the Albufera lagoon connecting with the open sea through a short drainage channel, oligohaline conditions are driven by flood gates preventing the entrance of seawater, that might also control the entrance of small sized crabs from the open sea. Further SIBER results indicated an overlap of 32% between isotopic niches, that support the undergoing of certain segregation between both investigated size classes. Additionally, 20% higher values of TP were also detected in subadults from the Alfacs Bay, in agreement with much higher values reported for mature females in the Open Sea (TP of 4.4; [49]), and the possible partial retention of the signals.

No significant effects of sex were observed for δ^13^C and δ^15^N at any single locality. Yet, for δ^13^C, there were inconsistent differences between large individuals of both sexes; large females showed more enriched signatures in Riumar, similar values to males in Encanyissada and Alfacs (Catalonia), Perelló and Pujol (Valencia), and Cola del Rio and Orones (Alicante), and more depleted signatures in Convenio (Alicante). Additionally, in the open sea (Valencia), only mature females were available, whereas they were absent in Mar Menor (Murcia) at the moment of sampling (but see [67]). Overall, these reduced patterns of differences are consistent with the large overlap in the isotopic niche between sexes (44%), and with the similar TP found for large individuals in the Encanyissada lagoon (M: 2.55 vs. F: 2.31) and in the Alfacs Bay (M: 2.27 vs. F: 2.28). Riumar, in the lower Ebro River (around 3 to 13 km from the mouth), might constitute one of the more similar environments to native areas (relatively large estuary with ample connectivity with the open sea) available in the Spanish Mediterranean. The salt wedge of the Ebro River stretches for ca. 27 km upstream [68], and allows for the distribution of organisms by salinity strata. Also, the salt wedge might favor the return of some females from the open sea with higher isotopic signatures [49], since some individuals can live up to 3 years, depending on environmental conditions and the health of the crab [24]. Additionally, salt wedge increase following recurrent recent droughts [65] (CHEbro, 2023) might have also favored the arrival of females. A somewhat unexpected dominance of large females (N = 17 individuals, whereas only 3 large males featuring similar isotopic patterns could be captured) was observed in Cola del Río located in the mouth of the Segura River (Alicante) at a salinity of around 5, which could be attributed to the proximity to the open sea (about 100 m) and to significant agricultural water discharge at the moment of sampling. Abundance patterns skewed towards the dominance of mature females in areas close to the open sea are commonly reported for native areas (e.g., [23, 69] and have also been observed in the Ebro Delta and in front of the Albufera in Valencia ([49]; this study). Yet, mature females do not seem to be equally attracted by areas of hypersaline waters (>40) areas such as the Mar Menor, as suggested by the absence of megalopae larvae in different years sampled with bongo nets and larval collectors (unpublished data), and largely variable sex ratios observed among quarters [67]. Large males usually dominate in extremely isolated freshwater localities such as the Orones channel (>10 km from the sea) because they do not migrate directionally along the salinity gradient [15]. Only in the Convenio channel connecting the Segura River with the Hondo lagoon, δ^13^C signatures of mature females were lower than those of large males, suggesting that few of them are able to reach the locality from other more oligohaline environments through the irrigation network. However, most of the localities featuring less extreme connectivity in terms of isolation and/ or distance to the sea did not show significant differences in isotopic and abundance patterns by sex, pointing to a more homogeneous environment with regards to salinity [20], particularly in deeper waters (> 0.75 m; [66]).

To conclude, low salinities, often caused by agricultural freshwater discharge and associated eutrophication was a central variable shaping the stable isotope composition (both δ^13^C and δ^15^N) of blue crab and dietary resources (mostly δ^15^N, except for δ^13^C in plants) along the Spanish Mediterranean coast. Its effect was mostly detected at the spatial scale of region and locality, featuring contrasting salinities (particularly between Murcia and Alicante) whereas individual aspects of sex and size displayed comparably lower effects. Compared to typical distances travelled in and out of estuaries by early stages and gravid females, which can exceed 200 km [23], the most remote locations in Alicante were only 10–15 km from the sea and displayed greater changes and clustering in isotopic values than expected given the short distances. However, entrance to estuarine habitats may be affected by human activities such as the presence of regulated channels, elevated agricultural drainage flow, or reduced river discharge increasing the entrance of the salt wedge, which occurred in most of our study areas and may lead to enhanced isotopic signatures in smaller individuals compared to previous studies ([61, 62] vs. this study). Additionally, confined and semi-confined water masses might display more homogeneous salinity features [20], thus concealing sex patterns between large individuals. Overall, we found a comprehensible agreement between stable isotope patterns across the salinity gradient, and isotopic niche assessment. However, dietary evaluation with stable isotope mixing models often provided wide credible intervals and ambiguous results due to the complex nature of isotopic and fractionation data (see also [70]. For instance, [71] demonstrated the limitations of using stable isotope mixing models to accurately determine the diet of Arctic Peregrine Falcon nestlings, showing that camera monitoring provided more precise diet estimates compared to isotope analysis alone. When possible, we advise the estimation of TP as a reliable alternative for comparatively assessing the local use of food resources [30]. For blue crab, more time-consuming, traditional methods such as stomach contents analyses still provide detailed information about dietary items [72] and could be used to verify results derived from mixing models.

## Supporting information

S1 Dataset Diet species at each locality. (XLSX)

S2 Dataset Ellipses overlap by factor. (XLSX)

## References

[1] Zenetos A, Çinar ME, Pancucci-Papadopoulou MA, Harmelin JG, Furnari G, Andaloro F, et al. Annotated list of marine alien species in the Mediterranean with records of the worst invasive species. Mediterr Mar Sci. 2005; 6 (2):63–118.

[2] Nehring S. Invasion history and success of the american blue crab Callinectes sapidus in European and adjacent waters In: Galil BS, Clark PF, Carlton JT, Editors. In the wrong place-alien marine crustaceans: distribution, biology and impacts. Invading Nature-Springer Series 6. 2011. pp. 607–624.

[3] Mancinelli G, Raho D, Zotti M, Alujević K, Guerra MT, Vizzini S. Trophic flexibility of the Atlantic blue crab Callinectes sapidus in invaded coastal systems of the Apulia region (SE Italy): A stable isotope analysis. Estuar Coastal Shelf Sci. 2017; 198:421–431.

[4] López V, Rodon J. 2018 [cited 9 Jul 2024]. Diagnosi i situació actual del cranc blau (Callinectes sapidus) al Delta de l’Ebre. Direcció General de Pesca i Afers Marítims. Generalitat de Catalunya, pp. 1–86 [Internet]. Available from: https://agricultura.gencat.cat/ca/detalls/Publicacio/2018-diagonsi-i-situacio-actual-cranc-blau-al-delte-ebre

[5] Hill J, Fowler DL, Avyle MV. 1989 [cited 9 Jul 2024]. Species profiles: Life histories and environmental requirements of coastal fishes and invertebrates (Mid-Atlantic). Blue crab US Army Corps of Engineers Report No, TR-EL-82-4/82(11.100), pp. 18 [Internet]. Available from: https://www.osti.gov/biblio/5907707.

[6] Serbetis C. Un nouveau crustacé commestible en mer Egeé Callinectes sapidus Rath(Decapod brach). Proc Gen Fish Counc Medit. 1959; 5:505–507.

[7] Mancinelli G, Bardelli R, Zenetos A. A global occurrence database of the Atlantic blue crab *Callinectes sapidus*. Scientific data. 2021; 8(1):111. 33863897 10.1038/s41597-021-00888-w PMC8052346

[8] Castejón D, Guerao G. A new record of the American blue crab, *Callinectes sapidus* Rathbun, 1896 (Decapoda: Brachyura: Portunidae), from the Mediterranean coast of the Iberian Peninsula. BioInv Rec. 2013; 2:141–143.

[9] González-Wanguemert M, Pujol JA. First record of the Atlantic blue crab *Callinectes sapidus* (Crustacea: Brachyura: Portunidae) in the Segura river mouth (Spain, southwestern Mediterranean Sea). Turkish J Zool. 2016; 40(4):615–619.

[10] Casalduero FG, Esplá AR, Muñoz AI, Castillo FG, Hernández FM, González-Carrión F. Allochthonous marine invertebrates in the Menor Mar lagoon. In: Instituto Español de Oceanografía, Editor. Mar Menor: una laguna singular y sensible. Evaluación científica de su estado. 2016, pp. 157–178.

[11] García L, Pinya S, Colomar V, París T, Puig M, Rebassa, et al. The first recorded occurrences of the invasive crab *Callinectes sapidus* Rathbun, 1896 (Crustacea: Decapoda: Portunidae) in coastal lagoons of the Balearic Islands (Spain). BioInvasions Rec. 2018; 7(2):191–196.

[12] Vasconcelos P, Carvalho AN, Piló D, Pereira F, Encarnação J, Gaspar MB, Teodósio MA. Recent and consecutive records of the Atlantic blue crab (*Callinectes sapidus* Rathbun, 1896): rapid westward expansion and confirmed establishment along the Southern Coast of Portugal. Thalassas. 2019; 35(2):485–494.

[13] González-Ortegón E, Berger S, Encarnação J, Chairi H, Morais P, Teodósio MA, et al. Free pass through the pillars of Hercules? Genetic and historical insights into the recent expansion of the Atlantic blue crab *Callinectes sapidus* to the West and the East of the Strait of Gibraltar. Front Mar Sci. 2022; 9:918026.

[14] Hill JM, Weissburg MJ. Habitat complexity and predator size mediate interactions between intraguild blue crab predators and mud crab prey in oyster reefs. Mar Ecol Progr Ser. 2013; 488:209–219.

[15] Hines AH. Ecology of juvenile and adult blue crabs, In: Kennedy VS, Cronin LE, Editors. The Blue Crab: *Callinectes sapidus*. Maryland Sea Grant College. 2007; pp. 565–654.

[16] Prado P, Ibáñez C, Chen L, Caiola N. Feeding habits and short-term mobility patterns of blue crab, *Callinectes sapidus*, across invaded habitats of the Ebro Delta subjected to contrasting salinity. Estuar Coasts. 2022; 45(3):839–855.

[17] Laughlin RA. Feeding habits of the blue crab, *Callinectes sapidus* Rathbun, in the Apalachicola estuary, Florida. Bullet Mar Sci. 1982; 32(4): 807–822.

[18] Stoner AW, Buchanan, BA (1990) Ontogeny and overlap in the diets of four tropical *Callinectes species* Bull Mar Sci 46:3–12

[19] Miller RE, Sulkin SD, Lippson RL. Composition and seasonal abundance of the blue crab, Callinectes sapidus Rathbun, in the Chesapeake and Delaware Canal and adjacent waters. Chesap Sci. 1975; 16:27–31.

[20] Ramach S, Darnell MZ, Avissar N, Rittschof D. Habitat use and population dynamics of blue crabs, *Callinectes sapidus*, in a high-salinity embayment. J Shellfish Res. 2009; 28(3):635–640.

[21] Schweitzer MD, Withers K. Size and distribution of blue crabs (*Callinectes sapidus*) with regard to salinity in the upper Nueces Estuary. Texas Gulf Mex Sci. 2009; 27(2):7

[22] Eggleston DB. Functional responses of blue crabs *Callinectes sapidus* Rathbun feeding on juvenile oysters *Crassostrea virginica* (Gmelin): effects of predator sex and size, and prey size. J Exp Mar Biol Ecol. 1990; 143(1–2):73–90.

[23] Aguilar R, Hines AH, Wolcott TG, Wolcott DL, Kramer MA, Lipcius RN. The timing and route of movement and migration of post-copulatory female blue crabs, *Callinectes sapidus* Rathbun, from the upper Chesapeake Bay. J Exp Mar Biol Ecol. 2015; 319(1–2):117–128.

[24] Millikin MR, Williams AB. 1984. [cited 2014 Jul 9]. Synopsis of biological data on the blue crab, *Callinectes sapidus* (Rathbun). NOAA Technical Report NMFS 1, FAO Fisheries Synopsis No 138 NOAA, National Marine Fisheries Service, pp. 1–39 [internet]. Available from: https://www.fao.org/4/ap942e/ap942e.pdf

[25] Gil-Fernández A, Rodilla M, Prado P, Falco S. Early life stages of the invasive Atlantic blue crab *Callinectes sapidus* in the Western Mediterranean Sea. Estuar Coast Shelf Sci. 2024; 296:108593.

[26] Jackson AL, Inger R, Parnell AC, Bearhop S. Comparing isotopic niche widths among and within communities: SIBER-Stable Isotope Bayesian Ellipses in R. J Anim Ecol. 2011; 80(3):595–602. doi: 10.1111/j.1365-2656.2011.01806.x 21401589

[27] Jackson A, Parnell A, Jackson MA. Package ‘SIBER’ R package version. 2019; 2(4).

[28] Stock B, Semmens B. 2016 [cited 2014 Jul 9]. MixSIAR GUI user manual v3 1. Scripps Institution of Oceanography, UC San Diego, California, USA [Internet]. Available from: http://cran.nexr.com/web/packages/MixSIAR/README.html

[29] Stock BC, Jackson AL, Ward EJ, Parnell AC, Phillips DL, Semmens BX. Analyzing mixing systems using a new generation of Bayesian tracer mixing models. PeerJ, 2018; 6:e5096. doi: 10.7717/peerj.5096 29942712 PMC6015753

[30] Post DM. Using stable isotopes to estimate trophic position: models, methods, and assumptions. Ecology. 2002; 83(3):703–718.

[31] Estiarte M, Peñuelas J, López-Martínez C, Pérez-Obiol R. Holocene palaeoenvironment in a former coastal lagoon of the arid south eastern Iberian Peninsula: salinization effects on δ15N. Veg Hist Archaeobotany. 2008; 17:66ṅ7e674.

[32] Obrador B, Pretus JL. Budgets of organic and inorganic carbon in a Mediterranean coastal lagoon dominated by submerged vegetation. Hydrobiologia. 2012; 669(1):35e54.

[33] Deegan LA, Garritt RH. Evidence for spatial variability in estuarine food webs. Mar Ecol Prog Ser. 1997; 147:31–47.

[34] Vizzini S, Savona B, Chi TD, Mazzola A. Spatial variability of stable carbon and nitrogen isotope ratios in a Mediterranean coastal lagoon. Hydrobiologia. 2005; 550:73e82.

[35] Wozniak AS, Roman CT, Wainright SC, McKinney, James-Pirri MJ. Monitoring food web changes in tide-restored salt marshes: a carbon stable isotope approach. Estuar Coasts. 2006; 29:568–578.

[36] Prado P, Vergara C, Caiola N, Ibáñez C. Influence of salinity regime on the food-web structure and feeding ecology of fish species from Mediterranean coastal lagoons. Estuar Coast Shelf Sci. 2014; 139:1–10.

[37] Fantle MS, Dittel AI, Schwalm SM, Epifanio CE, Fogel ML. A food web analysis of the juvenile blue crab, *Callinectes sapidus*, using stable isotopes in whole animals and individual amino acids. Oecologia. 1999; 120:416–426. 28308018 10.1007/s004420050874

[38] Di Muri C, Rosati I, Bardelli R, Cilenti L, Veli DL, Falco S, et al. An individual-based dataset of carbon and nitrogen isotopic data of *Callinectes sapidus* in invaded Mediterranean waters. Biodiv Data J, 2022; 10:e77516. 10.3897/BDJ.10.e77516 PMC8807565 35115881

[39] Colmenarejo MF, Sánchez E, Borja R, Travieso L, Cirujano S, Echevarrias JL, et al. Evaluation of the quality of the water in El Hondo Natural Park located in the east of Spain. J Environ Sci Health. 2007; Part A 42(7):969–981. 10.1080/10934520701370378 17558777

[40] Urquijo J, De Stefano L. Perception of drought and local responses by farmers: a perspective from the Jucar River Basin, Spain. Water Resour Manag. 2016; 30:577–591.

[41] Álvarez-Rogel J, Barberá GG, Maxwell B, Guerrero-Brotons M, Díaz-García C, Martínez-Sánchez, et al. The case of Mar Menor eutrophication: State of the art and description of tested Nature-Based Solutions. Ecol Engin. 2020; 158:106086.

[42] McCann MJ, Jensen OP. 2018 [cited 2014 Jul 9]. Laboratory experiments to determine trophic enrichment factors of stables isotope and fatty acid biomarkers in the blue crab *Callinectes sapidus*. Gulf of Mexico Research Initiative Information and Data Cooperative (GRIIDC), Harte Research Institute, Texas A&M University–Corpus[internet]. Available from: https://data.griidc.org/data/R4.x264.221:0003. doi: 10.7266/N76971K2

[43] Post DM, Layman CA, Arrington DA, Takimoto G, Quattrochi J, Montana GC. Getting to the fat of the matter: models, methods and assumptions for dealing with lipids in stable isotope analyses. Oecologia. 2007; 152(1):179–189. doi: 10.1007/s00442-006-0630-x 17225157

[44] Prado P, Carmichael RH, Watts SA, Cebrian J, Heck KL Jr. Diet-dependent δ^13^C and δ^15^N fractionation among sea urchin *Lytechinus variegatus* tissues: implications for food web models. Mar Ecol Progr Ser. 2012; 462:175–190.

[45] Mancinelli G, Glamuzina B, Petrić M, Carrozzo L Glamuzina L, Zotti M, et al. The trophic position of the Atlantic blue crab *Callinectes sapidus* Rathbun 1896 in the food web of Parila Lagoon (South Eastern Adriatic Croatia): A first assessment using stable isotopes. Mediterr Mar Sci. 2016; 17: 634–643.

[46] Mancinelli G, Carrozzo L, Marini G, Costantini ML, Rossi L, Pinna M. Occurrence of the Atlantic blue crab *Callinectes sapidus* Rathbun, 1896 in two Mediterranean coastal habitats: Temporary visitor or permanent resident?. Estuar Coastal Shelf Sci. 2013; 135:46–56.

[47] Carrozzo L, Potenza L, Carlino P, Costantini M L, Rossi L, Mancinelli G. Seasonal abundance and trophic position of the Atlantic blue crab *Callinectes sapidus* Rathbun 1896 in a Mediterranean coastal habitat. Rendiconti Lincei. 2014; 25:201–208.

[48] Aslan H, Polito MJ. Trophic ecology of the Atlantic blue crab *Callinectes sapidus* as an invasive non-native species in the Aegean Sea. Biol Inv. 2021; 23:2289–2304.

[49] Prado P, Baeta M, Mestre E, Solis MA, Sanhauja I, Gairin I, Camps-Castellà J, Falco S, Ballesteros M. Trophic role and predatory interactions between the blue crab, *Callinectes sapidus*, and native species in open waters of the Ebro Delta. Estuar Coastal Shelf Sci. 2024; 298:108638

[50] Chouvelon T, Spitz J, Caurant F, Mèndez-Fernandez P, Chappuis A, Laugier F, et al. Revisiting the use of δ15N in meso-scale studies of marine food webs by considering spatio-temporal variations in stable isotopic signatures-The case of an open ecosystem: The Bay of Biscay (North-East Atlantic). Progr Oceanogr. 2012; 101(1):92–105.

[51] Vizzini S, Mazzola A. Stable isotope evidence for the environmental impact of a land-based fish farm in the western Mediterranean. Mar Pollut Bull. 2004;49: 61e70. doi: 10.1016/j.marpolbul.2004.01.008 15234874

[52] Gilabert J. Seasonal plankton dynamics in a Mediterranean hypersaline coastal lagoon: the Mar Menor. J Plankton Res. 2001; 23(2):207–218.

[53] Vizzini S, Mazzola A. Seasonal variations in the stable carbon and nitrogen isotope ratios (13C/12C and 15N/14N) of primary producers and consumers in a western Mediterranean coastal lagoon. Mar Biol. 2003; 142:1009e1018.

[54] Ruiz-Fernández JM, Belando-Torrentes MD, Bernardeau-Esteller J, Mercado-Carmona JM. Mar Menor lagoon: an iconic case of ecosystem collapse. Harmful Algae News, Unesco. 2022; 70:1–5. Available from: https://habioc-unescoorg/

[55] Day JW, Maltby E, Ibáñez C. River basin management and delta sustainability: A commentary on the Ebro Delta and the Spanish National Hydrological. Plan Ecol Eng. 2006; 26(2):85–99.

[56] Calvo S, Romo S, Soria J, Picó Y. Pesticide contamination in water and sediment of the aquatic systems of the Natural Park of the Albufera of Valencia (Spain) during the rice cultivation period. Sci Tot Environ. 2021; 774:145009.

[57] Cole ML, Valiela I, Kroeger KD, Tomasky GL, Cebrian J, Wigand C, et al. Assessment of a δ^15^N isotopic method to indicate anthropogenic eutrophication in aquatic ecosystems. J Environ Qual. 2004; 33(1):124–132. 14964366 10.2134/jeq2004.1240

[58] Wyatt AS, Waite AM, Humphries S. Variability in isotope discrimination factors in coral reef fishes: implications for diet and food web reconstruction. PLoS One. 2010:5(10):e13682. doi: 10.1371/journal.pone.0013682 21060681 PMC2965116

[59] Martin D, Pinedo S, Sardá R. Distribution patterns and trophic structure of soft-bottom polychaete assemblages in a north-western Mediterranean shallow-water bay. Ophelia. 2000; 53(1)1–17.

[60] Mäkelä A, Witte U, Archambault P. Benthic macroinfaunal community structure, resource utilisation and trophic relationships in two Canadian Arctic Archipelago polynyas. PloS one. 2017; 12(8):e0183034. doi: 10.1371/journal.pone.0183034 28850574 PMC5574606

[61] Hoeinghaus DJ, Davis SE III. Size-based trophic shifts of saltmarsh dwelling blue crabs elucidated by dual stable C and N isotope analyses. Mar Ecol Progr Ser. 2007; 334:199–204.

[62] Dittel AI, Epifanio CE, Fogel ML. Trophic relationships of juvenile blue crabs (*Callinectes sapidus*) in estuarine habitats. Hydrobiologia, 2006; 568:379–390.

[63] deFur PL, Nusbaumer D, Lewis RJ. Physiological aspects of molting in blue crabs from the tidal fresh-water Potomac River, Virginia. J Crust Biol. 1988; 8:12–19.

[64] Rozas LP Odum WE. Use of tidal freshwater marshes by fishes and macrofaunal crustaceans along a marsh stream-order gradient. Estuaries. 1987; 10:36–43.

[65] CHEbro (Confederación Hidrográfica del Ebro); 2023 [cited 2014 Jul 9]. Plan de Sequía 2023 [Internet]. Available from: https://www.chebro.es/en-GB/plan-de-sequ%C3%ADa-2023

[66] Cerralbo P, Espino M, Grifoll M, Valle-Levinson A. Subtidal circulation in a microtidal Mediterranean bay. Sci Mar. 2018; 82(4):231–243.

[67] Guijarro-García E, Vivas M, García E, Barcala E, Trives M, Muñoz A. 2019, Sep 9–12 [cited 9 Jul 2024]. Atlantic blue crab (Callinectes sapidus Rathbun, 1896) in a protected coastal lagoon in SE Spain. In: Front Mar Sci Conference Abstract: XX Iberian Symposium on Marine Biology Studies (SIEBM XX) [Internet]. Available from: https://www.frontiersin.org/10.3389%2Fconf.fmars.2019.08.00196/event_abstract.

[68] Ibáñez C, Caiola N, Belmar O. Environmental flows in the lower Ebro River and Delta: Current status and guidelines for a holistic approach. Water. 2020; 12(10):2670.

[69] Archambault JA, Wenner EL, Whitaker JD. Life history and abundance of blue crab, *Callinectes sapidus* Rathbun, at Charleston Harbor, South Carolina. Bull Mar Sci. 1990; 46(1):145–158.

[70] Osada Y, Matsubayashi J, Tayasu I.Diagnosing underdetermination in stable isotope mixing models. Plos one. 2021; 16(10):e0257818. doi: 10.1371/journal.pone.0257818 34597310 PMC8486109

[71] Robinson BG, Franke A, Derocher AE. Stable isotope mixing models fail to estimate the diet of an avian predator. Auk. 2018; 135(1):60–70.

[72] Öndes F, Esteso I, Guijarro-García E, Barcala E, Giménez F, Ramos-Esplá AA, et al. 2024. [cited 2014 Jul 14]. Feeding habits of the invasive atlantic blue crab, *Callinectes sapidus*, in different habitats of the Southeastern Iberian Peninsula. IX International Symposium on Marine Sciences Valencia (Spain), 10-12th July 2024.

